# Correcting the Bias of Empirical Frequency Parameter Estimators in Codon Models

**DOI:** 10.1371/journal.pone.0011230

**Published:** 2010-07-30

**Authors:** Sergei Kosakovsky Pond, Wayne Delport, Spencer V. Muse, Konrad Scheffler

**Affiliations:** 1 Department of Medicine, University of California San Diego, San Diego, California, United States of America; 2 Department of Pathology, University of California San Diego, San Diego, California, United States of America; 3 Department of Statistics, North Carolina State University, Raleigh, North Carolina, United States of America; 4 Computer Science Division, Department of Mathematical Sciences, Stellenbosch University, Stellenbosch, South Africa; Aarhus University, Denmark

## Abstract

Markov models of codon substitution are powerful inferential tools for studying biological processes such as natural selection and preferences in amino acid substitution. The equilibrium character distributions of these models are almost always estimated using nucleotide frequencies observed in a sequence alignment, primarily as a matter of historical convention. In this note, we demonstrate that a popular class of such estimators are biased, and that this bias has an adverse effect on goodness of fit and estimates of substitution rates. We propose a “corrected” empirical estimator that begins with observed nucleotide counts, but accounts for the nucleotide composition of stop codons. We show via simulation that the corrected estimates outperform the *de facto* standard 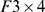 estimates not just by providing better estimates of the frequencies themselves, but also by leading to improved estimation of other parameters in the evolutionary models. On a curated collection of 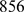 sequence alignments, our estimators show a significant improvement in goodness of fit compared to the 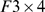 approach. Maximum likelihood estimation of the frequency parameters appears to be warranted in many cases, albeit at a greater computational cost. Our results demonstrate that there is little justification, either statistical or computational, for continued use of the 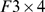-style estimators.

## Introduction

**Figure 1:**
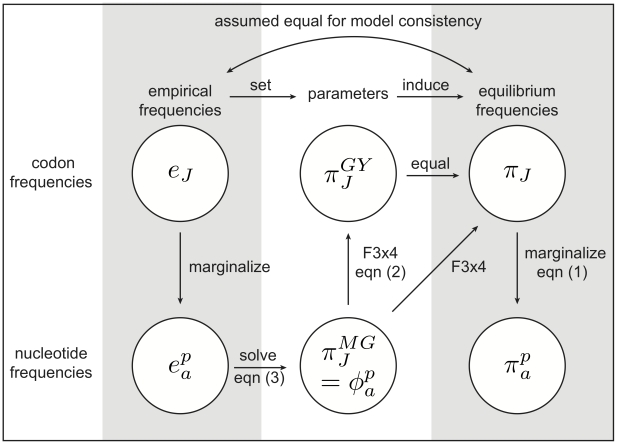
Relationships between empirical frequencies, frequency parameters and equilibrium frequencies in codon models.

Virtually all codon models in wide use today (see [1], [2] for recent reviews) are members of the class of finite-state, continuous time reversible Markov chains, each defined by an instantaneous rate matrix 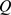. Transition matrices for finite amounts of time are found via the matrix exponential of 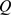, so the probability that a position initially occupied by codon 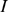 is occupied by codon 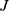 after 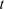 units of time is 
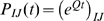
 (throughout the manuscript we will use upper-case letters to index codons and lower-case letters to index nucleotides). If 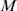 is a model in this class, the individual entries of its rate matrix can be written in the canonical form 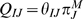. The 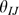 can be thought of as “rate parameters” that govern the relative rates of substitutions between different codons, while parameters 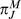 induce the equilibrium frequencies of the codons. The choice of 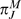 is the primary distinction between the two popular families of codon models: MG (introduced in [3]) and GY (introduced in [4]). How to best estimate the 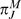
— or more precisely, how to estimate model parameters that actually determine the 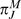
— from sequence alignments is the focus of this note. In order to frame this discussion we need to define what we mean by empirical *frequencies*, model *parameters* and *equilibrium* frequencies (Figure 1). Given an observed alignment, the position-specific empirical nucleotide frequencies, 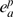 where 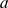 is a nucleotide (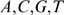) and 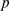 the codon position (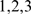), can be estimated directly by counts from the data, and the empirical codon frequencies, 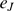, can be estimated by counts as well (the latter gives rise to the F61 codon frequency estimator [4]). Either of these estimates can be used to set model parameters, however typical alignments have insufficient information for the direct estimation of empirical codon frequencies with a sufficient degree of confidence. Rather, the empirical nucleotide frequencies are used to set the nucleotide frequency parameters, 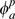, and by multiplication of their constituents, the codon frequency parameters, 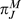. For example, in the original MG94 model of codon evolution [3], the equilibrium frequency of codon 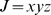 is given by 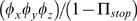, where 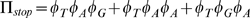. A common extension of this model, referred to as MG94 F3×4, allows the three codon positions to have their own nucleotide frequency parameters and leads to equilibrium codon expressed as:
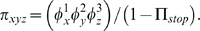
(1)In this expression the superscripts indicate the position, and the equation for 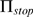 is modified in the obvious way. If we set all of the model nucleotide frequency parameters to be equal, i.e. 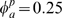, the result is equal equilibrium frequencies for all codons, i.e. 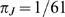 for all 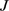. This vector of codon equilibrium frequencies allows us to easily tabulate, via marginalization, the equilibrium frequencies of each nucleotide at each position:
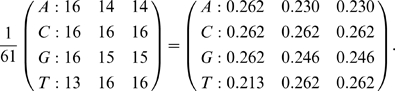
(2)


Note that there are only 13 occurrences of *T* in the first position, 14 of *A* in the second position, etc because the model explicitly disallows (*TAG*,*TAA*,*TGA*) as is standard for all other codon models. The finding from this exercise is that when one sets all the 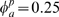, each of the codon equilibrium frequencies, 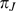 takes the anticipated value of 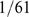. However, remarkably, the equilibrium nucleotide frequencies generated by this model are *not* the anticipated 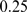. For instance, the equilibrium frequency of 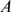 at the first position is 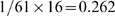. Traditionally, the empirical nucleotide frequencies are used to set nucleotide frequency parameters, and it is therefore assumed that the induced equilibrium nucleotide frequencies are equal to those observed in the alignment. However, given that the nucleotide composition of stop codons is not accounted for, this practice is flawed, because 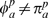. The conflation of frequency parameters (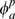) and equilibrium nucleotide (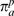) frequencies results in incorrect estimates of equilibrium nucleotide (and codon) frequencies as demonstrated in (2) above. This phenomenon is not restricted to the MG family of models. It is simple to demonstrate the exact same behavior for the GY family of models, again because of the incorrect designation of nucleotide frequency parameters in the rate matrix as equal to empirical nucleotide frequencies. We show that the traditional identification of frequency parameters and observed nucleotide frequencies leads to a cascade of problems. Model frequency parameters are estimated with bias, which leads to biased estimation of the equilibrium codon frequencies, which leads to compensatory biased estimation of the substitution rate parameters. We propose a correction, and a maximum likelihood frequency parameterization and show that both these approaches are not similarly biased, and therefore advocate their use in codon models.

## Materials and Methods

To ensure clarity of presentation, we first carefully introduce the necessary notation (summarized in Figure 1). For a given substitution model, let 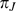 be the frequency of sense codon 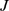 (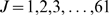) in its equilibrium distribution, and 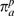, 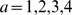 be the equilibrium frequency of nucleotide 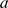 in codon position 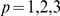. When necessary, we will indicate specific models via a superscript (ie, MG or GY). The position specific nucleotide equilibrium frequencies, 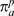, are uniquely determined by the codon equilibrium frequencies, 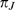, through marginalization, e.g. 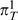 is simply the sum of frequencies of the 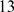 sense codons that have a T in their first position, e.g. as in equation (2).

These equilibrium frequencies, of both nucleotides and codons, have traditionally been assumed equal to empirical frequencies observed in a sequence alignment, 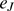 or 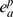, and used to set model parameters. If the specified model is correct, 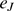 converges to 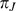 and 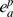 to 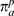 as the sequence length 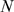 increases. (However, note that this result requires that the evolutionary process itself be at equilibrium; many important biological mechanisms— notably directional positive selection— are likely to disrupt equilibrium; see [5]–[7]).

Because the simple example in equation (2) demonstrated that the empirical and equilibrium nucleotide frequencies are not synonymous, we strive to obtain an expression that relates the equilibrium nucleotide frequencies to the model nucleotide frequencies, 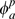, and through extension –to the observed empirical frequencies. Even though the MG and GY models treat equilibrium codon frequencies differently, it is a fortunate coincidence that in either case the 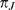 have identical forms when written in terms of 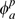. Given twelve MG nucleotide frequency parameters, only 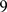 of which are independent because 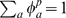 for each position 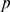, the equilibrium frequency of codon 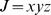 induced by their values is as in equation (1).

By using 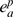 to directly estimate 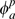 in equation (1), one obtains the popular 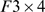 estimator of codon equilibrium frequencies – by far the most common estimator used in literature for both MG and GY classes of models. The statistical and computational appeal of 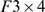 lies in its use of only 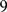 nucleotide parameters to describe 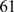 codon frequencies. However, the key shortcut— direct estimation of nucleotide frequency *parameters* with empirical nucleotide *frequencies* from the data— is flawed. The empirical nucleotide frequencies *are* unbiased estimates of the true equilibrium frequencies; unfortunately, the model parameters they are being used to estimate are something different. Thus, a fundamental problem with current practices is that use of the 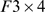 estimators with either MG or GY models leads to biased estimates of the 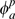, and in turn the 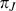. As we will show below, the problems do not end there, and lead to biased estimation of other model parameters.

We first present two approaches for correcting these estimation errors. The obvious, but more computationally demanding method is to estimate the 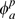 by maximum likelihood along with other model parameters. We dub this approach 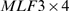. Theory suggests that estimates from this methodology will have all the desirable properties of maximum likelihood estimation. Maximum likelihood estimation of these values has been available in some software packages, e.g. in HyPhy [8], for a number of years, but to our knowledge it has rarely been used.

The second strategy, described here for the first time, relies on finding an expression for the induced equilibrium frequency of nucleotide 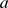 at codon position 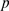 (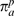) as a function of 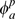. Since the 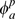 define codon equilibrium frequencies (equation 1), we can readily obtain such equations by marginalization:
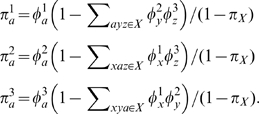
(3)


Here, 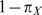 is simply scaling for the absence of stop codons: 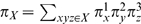, and 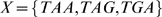 defines the set of stop codons. The *corrected* 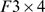, or 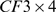 estimator equates 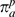 with observed nucleotide frequencies 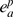, and then solves the nonlinear system (3) for 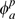 to obtain estimates of the latter. Because 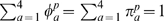, the above system of 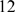 nonlinear equations relate 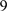 independent observed statistics (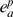, *e.g.* for 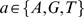) with 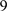 independent model parameters 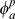. We were unable to obtain a closed form solution to the system, but it can be easily solved numerically at a negligible computational cost.

We conducted simulations to further investigate the effects of biases in the equilibrium frequencies on parameters typically estimated using phylogenetic models. We generated two-sequence codon alignments with uniform codon frequency composition (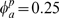). We used 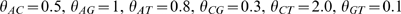 as substitution bias parameters in the MG94xREV model [9], and set the nonsynonymous/synonymous substitution rate ratio 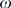 to 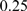. The two sequences were 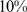 divergent on average, and the length of the alignment, 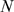, was one of 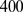, 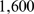 or 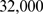 codons. 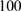 replicates were generated for each value of 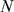. We compared the fits of 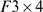, 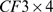 and 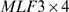 on simulated data sets, and furthermore compared simulated to inferred parameter estimates with each of the three frequency parameterizations. In addition to the simulated data, we fitted all three frequency parameterizations to a sample of 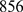 alignments from the carefully curated Pandit database [10]. All alignments were chosen to contain between 10 and 20 sequences and at least 200 reliably aligned codon sites. Given that each estimator has the same number of independent parameters (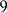), an improvement in log-likelihood under one of the models is considered as evidence in favor of the better fitting model, *e.g.* under the BIC [11] criterion. All new estimators for the MG94 class of models are implemented in HyPhy.

## Results and Discussion

**Figure 2:**
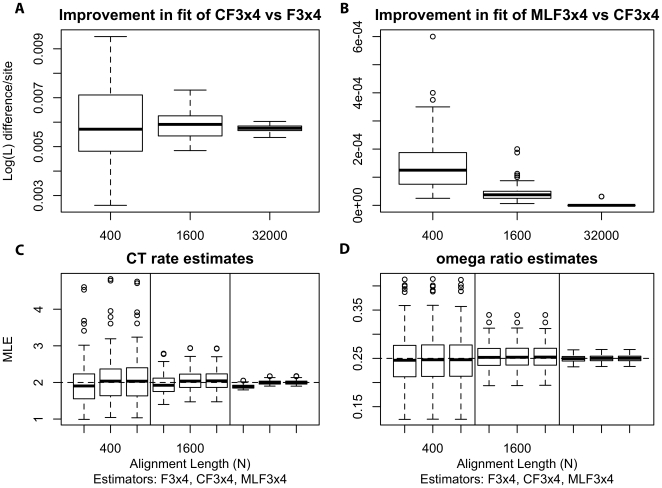
Comparison of frequency parameterizations fitted to simulated alignments. The top row (A,B) shows the comparison of 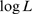 scores on simulated data obtained with different corrected frequency estimates; C) Bias in the estimate of the substitution rate 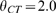 in near-asymptotic regime (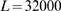) is apparent under 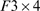, but does not exist for the other two estimators; D) variance of the 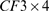 estimate for 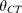 is reduced with increasing sample size.

We simulated data with a uniform codon frequency composition and fitted all three frequency parameterizations for alignments of various sequence lengths. The suboptimal nature of the 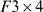 estimator is immediately apparent from Figure 2a, where the improvement in 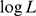 scores of the model equipped with the corrected estimator 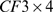 is shown. For all replicates, the 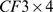 estimator yielded better 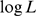, with median improvements of 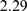, 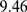, and 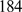 (for 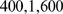, and 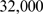 codons respectively), or approximately 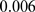 likelihood points per codon site. Note that as the sample size increased, the estimators from (3) effectively matched the performance of the maximum likelihood estimator (Figure 2b). Even more importantly, the use of the 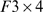 frequency estimator led to biased inference of other model parameters. Maximum likelihood estimates of some substitution rates were biased under the 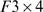, and the bias was progressively more pronounced with increasing sample size (Figure 2c). Indeed, for 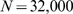, a simple likelihood ratio test rejected the (true) null of 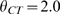 at 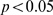 for all 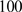 replicates. Biased MLEs of the substitution rate parameter 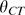 is a result of the under/overestimates of 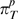 and 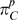 using 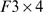. Similar results were seen for the other 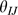. To our relief, the maximum likelihood estimate (MLE) for the 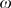 ratio was not noticeably affected even for the largest sample size (mean 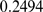, median 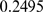, IQR 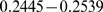 under 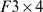; mean 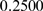, median 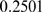, IQR 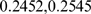 under 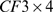, Figure 2d).

**Figure 3:**
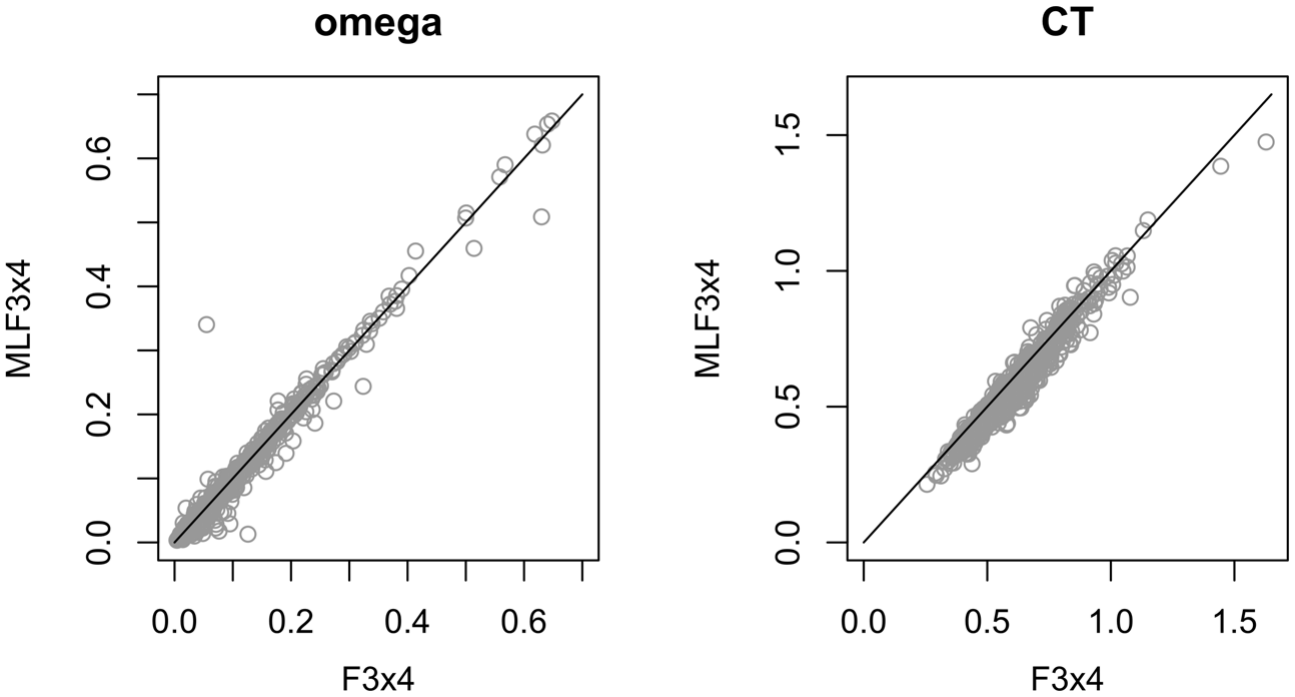
The effect of the frequency estimator on the inference of 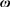 and 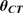 (relative to the 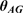 rate) substitution rate from 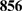 alignments sampled from the Pandit database [10]. The estimate of 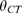 under 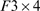 is biased downwards relative to 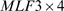.

For the Pandit alignments 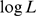 values were, of course, higher for the models estimated using 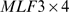 than for those using 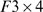. However, the magnitudes of the differences were impressive (median 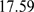, IQR 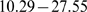, max 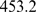). The 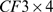 estimator improved the 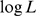 score of the 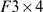 estimator for over 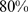
(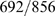) of the alignments by a median of 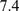 points; in the remaining cases the median decrease in 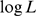 score was 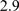 points. As with the simulated data, the MLEs of 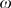 were largely unaffected by the choice of frequency estimators (but there were some datasets where the difference was large), while some substitution rate estimates appeared biased (Figure 3). For example, the estimates of 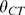 were strongly linearly correlated between 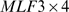 and 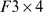 methods (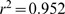), but the regression line was estimated as 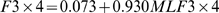, which recapitulates the downward bias observed on simulated data (if the estimates were unbiased, we would expect an intercept of zero and slope of one).

We have demonstrated through simulations that the almost universally used 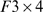 estimator of equilibrium frequencies in codon substitution models is biased, and we have pointed out how a misinterpretation of standard codon model parameters is responsible for these biases. Although this bias appears to have little effect on estimation of “composite” parameters such as the nonsynonymous/synonymous rate ratio (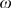) and branch lengths (results not shown), the bias has considerable damaging effects on the estimation of substitution rate parameters in the instantaneous rate matrix. This problem will become acutely relevant as researchers pursue finer-scale studies of the evolutionary process, such as developing substitution models with protein residue-dependent codon substitution rates [12], [13]. Since the computational burden of the 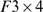 estimator is virtually identical to that of our proposed 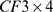 estimator, which in turn is only marginally faster than 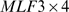, we recommend the use of either of the alternatives offered in this manuscript over the 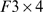 estimator. Our current recommendation is to obtain 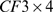 estimates and use them to initialize the optimization procedure for 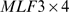 to speed up convergence.
