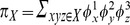# Correction: Correcting the Bias of Empirical Frequency Parameter Estimators in Codon Models

**DOI:** 10.1371/journal.pone.0106200

**Published:** 2014-08-19

**Authors:** The PLOS ONE Staff

There are errors in Equation (3). In the description of Equation (3), the equilibrium frequencies PIX are incorrectly defined in terms of pi, where in fact they should be defined in terms of phi. Please see the corrected description of Equation (3) here.